# Identification of Critical Genes and lncRNAs in Osteolysis after Total Hip Arthroplasty and Osteoarthritis by RNA Sequencing

**DOI:** 10.1155/2021/6681925

**Published:** 2021-03-13

**Authors:** Guang Yang, Kai Tang, Li Qiao, Yixin Li, Shui Sun

**Affiliations:** ^1^Department of Joint Surgery, Shandong Provincial Hospital Affiliated to Shandong First Medical University, Jinan 250000, China; ^2^Department of Orthopaedics, Shandong Provincial Hospital (Group) Ludong Hospital, Yantai 264000, China; ^3^Department of Orthopedics, Yantaishan Hospital, Yantai 264000, China; ^4^Department of Surgical Osteoarthritis, Jinan Central Hospital Affiliated to Shandong University, China

## Abstract

Total hip arthroplasty (THA) is a cost-effective treatment for osteoarthritis (OA), and osteolysis is a common complication of THA. This study was aimed at exploring the relevant molecular biomarkers for osteolysis after THA. We performed RNA sequence to identify and characterize expressed mRNAs and lncRNAs in OA and osteolysis. Differentially expressed mRNAs (DEmRNAs) and lncRNAs (DElncRNAs) in OA and osteolysis were acquired, as well as shared DEmRNAs/DElncRNAs in OA and osteolysis and osteolysis-specific DEmRNAs/DElncRNAs. Then, shared and osteolysis-specific DElncRNA-DEmRNA coexpression networks were constructed to further investigate the function of DElncRNAs and DEmRNAs in OA and osteolysis. In total, 343 DEmRNAs and 25 DElncRNAs in OA, 908 DEmRNAs and 107 DElncRNAs in osteolysis, and 406 DEmRNAs and 46 DElncRNAs between OA and osteolysis were acquired. A total of 136 shared DEmRNAs and 9 shared DElncRNAs in OA and osteolysis and 736 osteolysis-specific DEmRNAs and 103 osteolysis-specific DElncRNAs were acquired. Then, 128 shared DElncRNA-DEmRNA coexpression pairs and 522 osteolysis-specific DElncRNA-DEmRNA coexpression pairs were identified. The present study highlighted the roles of four interaction pairs, including two shared lncRNA-mRNA interaction pairs in OA and osteolysis (AC111000.4 and AC016831.6), which may function in the immune process of OA and osteolysis by regulating CD8A and CD8B, respectively, and two osteolysis-specific interaction pairs (AC090607.4-FOXO3 and TAL1-ABALON), which may play an important role in osteoclastogenesis.

## 1. Introduction

Osteoarthritis (OA) is a leading cause of chronic disability in old people. For the treatment, the total hip arthroplasty (THA) is a cost-effective way, which can reduce joint pain, restore joint function, and increase the quality of life of patients [1]. Despite the improvement of the quality of polyethylene, osteolysis remains a risk for older designs and younger, active patients. Osteolysis is a progressive, active, biologic cascade, a phenomenon due to a foreign body response to particulate wear debris from the prosthetic joint [2]. Increased wear particles activate osteoclast formation, and overweight osteoclasts caused much bone resorption, which eventually led to osteolysis [3]. Osteolysis, as a complication of THA, leads to prosthesis failure and bringing about additional suffering and burden for patients [4].

Long noncoding RNAs (lncRNAs), as a type of noncoding RNA, have been recognized as key regulatory molecules with diverse roles in gene expression, epigenetic modification, and protein activity [5]. Recently, lncRNA has been revealed to be involved in osteolysis. lncRNA TSIX was involved in particle-induced osteolysis by regulating miR-30a-5p to promote osteoblast apoptosis [6]. lncRNA DANCR inhibits osteoblast differentiation in osteolysis after THA through regulating FOXO1 [7]. lncRNA KCNQ1OT1 could ameliorate particle-induced osteolysis, by inhibiting miR-21a-5p to induce macrophage polarization [8]. However, more studies focused on the role of lncRNAs in osteolysis after THA need to be performed.

In this study, we, respectively, investigated the gene expression profiles of lncRNAs and mRNAs in patients with osteolysis after THA and OA, attempting to screen out differentially expressed mRNAs (DEmRNAs) and lncRNAs (DElncRNAs) associated with osteolysis after THA and OA. The objective of this study was to explore the underlying mechanism and the relevant molecular biomarkers for osteolysis after THA.

## 2. Materials and Methods

### 2.1. Subjects and Samples

The cohort subjected to RNA-Seq comprised 3 patients with OA, 3 patients with osteolysis after THA, and 3 healthy individuals. Inclusion criteria for patients with osteolysis after THA: (1) with current radiographic evidence of osteolysis and (2) received a THA after failure to improve function and pain after at least 6 months of conservative treatment. Subjects were excluded if they had any history of inflammatory arthropathy or known secondary causes of hip arthritis such as trauma, avascular necrosis, or developmental or childhood hip disease. Subjects were also excluded if they had taken courses of immunosuppressant agents or bisphosphonates for a continuous period of greater than 6 months since THA. OA was diagnosed according to the criteria of the American College of Rheumatology. Healthy individuals with no personal or family history of OA, no symptoms or signs of OA, or any other type of arthritis, or any painful condition of the joints, were included as controls. The participants with history of joint diseases, including inflammatory arthritis (rheumatoid arthritis or any other autoimmune disease), posttraumatic or postseptic arthritis, poliomyelitis, and skeletal dysplasia, were excluded.  Table 1 described the characteristics of all these participants. All samples were collected after obtaining written informed consent from every participant. This study was approved by the Ethics Committee of Shandong Provincial Hospital (No. 2020-123) and performed in accordance with the Declaration of Helsinki. Peripheral whole blood (2.5 mL) drawn from each subject was collected in PAXgene® RNA blood tubes and stored at -80°C prior to processing. RNA isolation was performed with PAXgene blood RNA kit. RNA integrity and concentration were evaluated with an Agilent 2100 Bioanalyzer (Agilent RNA 6000 Nano Kit). Total RNA samples used in subsequent experiments fulfilled the following requirements: RNA integrity number (RIN) > 7.0 and 28S/18S ≥ 1. In brief, total RNA was subjected to ribosomal RNA (rRNA) removal using Ribo-Zero. A total amount of 3 *μ*g RNA was used for library preparation. Libraries for sequencing were constructed according to the manufacturer's protocol. The quality of the libraries was determined using an Agilent 2100 Bioanalyzer and ABI StepOnePlus Real-Time PCR System. Based on the BGIseq platform, sequencing was performed. The raw sequencing data were submitted to sequencing quality control by FastQC to assess whether they will be used for subsequent data analysis. Reads with low quality (adaptor sequences, sequences with a quality score < 20, and sequences with an *N* base rate of raw reads > 10%) were removed. Clean reads were aligned with the human reference genome, Ensemble GRCh38.p7.

### 2.2. Identification of DEmRNAs and Functional Annotation

StringTie software was used to compare the results to the known transcriptome and calculate the transcriptional abundance. Then, Ballgown was used for quantification of gene expression levels, as well as analysis of intergroup differential expression of genes. DESeq2 was applied to identify DEmRNAs in OA vs. control and osteolysis after THA vs. control with a *p* value < 0.05. DEmRNAs between OA and osteolysis after THA were obtained with a *p* value < 0.05 as well. Hierarchical clustering analysis of DEmRNAs was performed with R package “pheatmap.” Then, shared DEmRNAs in OA and osteolysis after THA and osteolysis-specific DEmRNAs (DEmRNAs in osteolysis after THA but no differences in OA) were further identified with Venny 2.1.0. The Database for Annotation, Visualization and Integrated Discovery (DAVID), which is a web-based tool, provides integrated solutions for the annotation and analysis of genome-scale datasets from high-throughput sequencing. DAVID 6.8 was used to perform GO and KEGG enrichment analysis for shared DEmRNAs in OA and osteolysis after THA and osteolysis-specific DEmRNAs. The lower the *p* value, the more significant are the GO term and the pathway. A value of *p* < 0.05 was considered to be represented statistically significant.

### 2.3. Identification of DElncRNAs

DESeq2 was applied to identify DElncRNAs in OA vs. control and osteolysis after THA vs. control with a *p* value < 0.05 and ∣log_2_FC | >1.5. DElncRNAs between OA and osteolysis after THA were obtained with a *p* value < 0.05 and ∣log_2_FC | >1.5 as well. Hierarchical clustering analysis of DElncRNAs was performed with R package “pheatmap.” Then, shared DElncRNAs in OA and osteolysis after THA and osteolysis-specific DElncRNAs (DElncRNAs in osteolysis after THA but no differences in OA) were further identified with Venny 2.1.0.

### 2.4. DElncRNA-DEmRNA Coexpression Network and Functional Annotation

The shared and osteolysis-specific DElncRNA-DEmRNA coexpression networks were constructed to further investigate the potential functions of lncRNAs and mRNAs in OA and osteolysis. Pearson correlation coefficients were calculated between the expression values of DElncRNAs and DEmRNAs. The pairs with ∣PCC | >0.8 and *p* < 0.01 in shared DElncRNA-DEmRNA pairs and pairs with ∣PCC | >0.95 and *p* < 0.01 in osteolysis-specific DElncRNA-DEmRNA pairs were defined as coexpressed DElncRNA-DEmRNA pairs, respectively. Then coexpressed networks were visualized by using Cytoscape. DAVID 6.8 was used to perform GO and KEGG enrichment analysis for DEmRNAs in shared and osteolysis-specific DElncRNA-DEmRNA coexpression network. A value of *p* < 0.05 was considered to be represented statistically significant.

### 2.5. Statistical Analysis

Statistical analyses were performed using R software v3.5.3 (R Foundation for Statistical Computing, Vienna, Austria). All tests were two-tailed, and *p* values < 0.05 were considered statistically significant. In addition, the GraphPad Prism Software, version 7.0, was used for the statistical analysis of experimental data. The results are expressed as means ± standard deviations (SDs). In addition, we used Shapiro-Wilk to test the normal distribution of data. However, the statistical analysis of clinical data showed that the data are not normal distribution. Therefore, we choose the Kruskal-Wallis test for statistical analysis. Pearson correlation coefficients were calculated between the expression values of DElncRNAs and DEmRNAs. *p* < 0.05 was considered to indicate a significant difference between the groups.

## 3. Results

### 3.1. Identification of DEmRNAs and Functional Annotation

**Figure 1:**
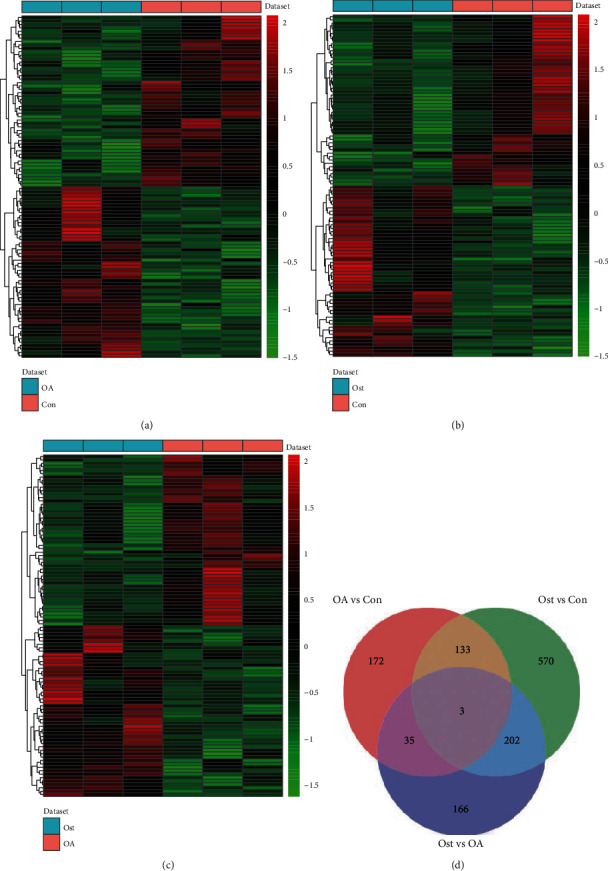
Identification of DEmRNAs: (a) heatmap of top 100 DEmRNAs between OA and normal controls; (b) heatmap of top 100 DEmRNAs between osteolysis after THA and normal controls; (c) heatmap of top 100 DEmRNAs between OA and osteolysis after THA; (d) Venn diagram of DEmRNAs in OA and osteolysis after THA. Con: controls; Ost: osteolysis.

DESeq2 was applied to identify DEmRNAs, and our results showed that a total of 343 DEmRNAs (184 up- and 159 downregulated) in OA vs. control, 908 DEmRNAs (429 up- and 479 downregulated) in osteolysis vs. control, and 406 DEmRNAs (112 up- and 294 downregulated) in OA vs. osteolysis were identified. Of these, HIST2H4A and CD8A, ZNF778 and TMEM56-RWDD3, and EPHB4 and MTURN were the most up- and downregulated DEmRNAs in OA vs. control, osteolysis vs. control, and OA vs. osteolysis, respectively (Table 2). The heatmap of the top 100 up- and downregulated DEmRNAs was shown in Figures 1(a)–1(c). The volcano plots of DEmRNAs are shown in Figures S1A–C. A total of 136 shared DEmRNAs (71 up- and 65 downregulated) in OA vs. control and osteolysis vs. control and 736 osteolysis-specific DEmRNAs (381 up- and 355 downregulated) were acquired (Figure 1(d)).

To investigate the functions of shared and osteolysis-specific DEmRNAs, DAVID 6.8 was used to perform GO and KEGG enrichment analysis. For shared DEmRNAs, cell activation (*p* = 2.81*E* − 03), leukocyte activation (*p* = 5.20*E* − 03), integral to plasma membrane (*p* = 3.17*E* − 04), and protein homodimerization activity (*p* = 1.93*E* − 02) were several significantly enriched GO terms, and mTOR signaling pathway (*p* = 1.89*E* − 02) and cell cycle (*p* = 3.81*E* − 02) were significantly enriched KEGG pathways (Figures S2, S4 A and B). For osteolysis-specific DEmRNAs, intracellular signaling cascade (*p* = 1.80*E* − 02), cell adhesion (*p* = 8.45*E* − 04), plasma membrane (*p* = 9.54*E* − 05), and cytoskeletal protein binding (*p* = 1.08*E* − 02) were several significantly enriched GO terms, and hematopoietic cell lineage (*p* = 1.35*E* − 02), cell adhesion molecules (CAMs) (*p* = 2.29*E* − 02), porphyrin and chlorophyll metabolism (*p* = 3.40*E* − 02), and systemic lupus erythematosus (*p* = 4.86*E* − 02) were significantly enriched KEGG pathways (Figures S3, S4 C–F).

### 3.2. Identification of DElncRNAs

**Figure 2:**
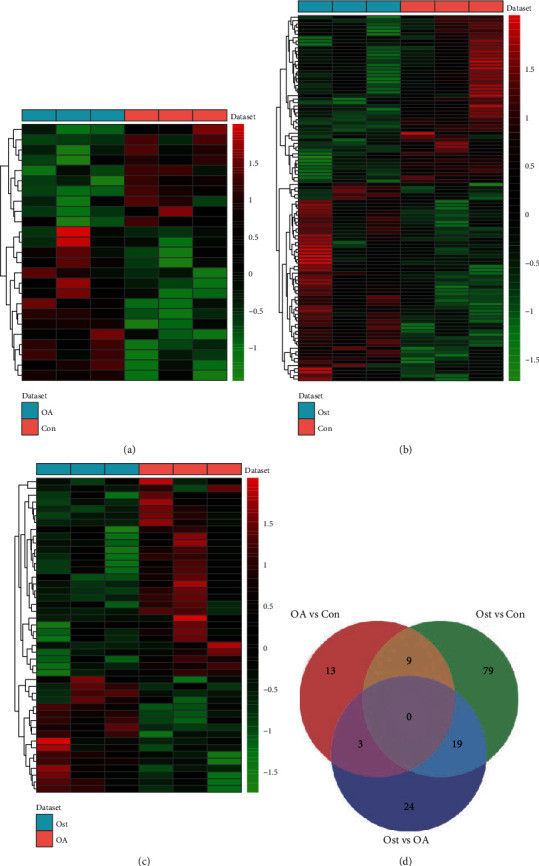
Identification of DElncRNAs: (a) heatmap of DElncRNAs between OA and normal controls; (b) heatmap of DElncRNAs between osteolysis after THA and normal controls; (c) heatmap of DElncRNAs between OA and osteolysis after THA; (d) Venn diagram of DElncRNAs in OA and osteolysis after THA. Con: controls; Ost: osteolysis.

DESeq2 was applied to identify DElncRNAs, and our results showed that a total of 25 DElncRNAs (15 up- and 10 downregulated) in OA vs. control, 107 DElncRNAs (58 up- and 49 downregulated) in osteolysis vs. control, and 46 DElncRNAs (17 up- and 29 downregulated) in OA vs. osteolysis were identified. Of these, NEAT1 and AC005726.2, FLJ42393 and AC123912.4, and AC016737.1 and AC123912.4 were the most up- and downregulated DElncRNAs in OA vs. control, osteolysis vs. control, and OA vs. osteolysis, respectively (Table 3). The heatmap of DElncRNAs is shown in Figures 2(a)–2(c). The volcano plots of DEmRNAs are shown in Figure S1D-F. A total of 9 shared DElncRNAs (6 up- and 3 downregulated) in OA vs. control and osteolysis vs. control and 103 osteolysis-specific DElncRNAs (52 up- and 51 downregulated) were acquired (Figure 2(d)).

### 3.3. DElncRNA-DEmRNA Coexpression Network and Functional Annotation

**Figure 3:**
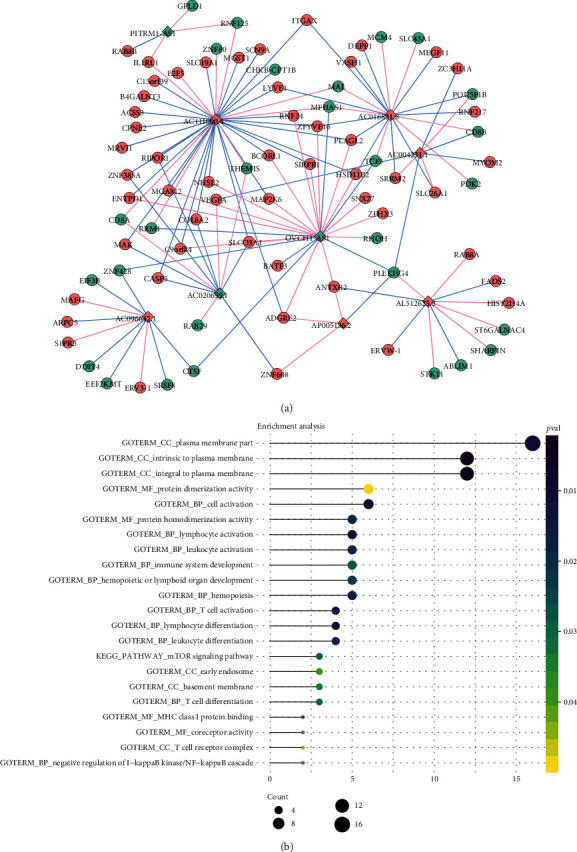
Shared DElncRNA-DEmRNA coexpression network and functional annotation. (a) Shared DElncRNA-DEmRNA co-expression network in OA and osteolysis after THA. The rhombus and ellipses represent DElncRNAs and DEmRNAs, respectively. Red and blue colors represent up- and downregulation, respectively. Red and blue edges represent positively and negatively correlated lncRNA-mRNA pairs, respectively. (b) Function enrichment.

To further investigate the potential functions of lncRNAs and mRNAs in OA and osteolysis, the shared and osteolysis-specific DElncRNA-DEmRNA coexpression networks were constructed. A total of 128 shared DElncRNA-DEmRNA coexpression pairs including 9 DElncRNAs and 81 DEmRNAs were obtained (Figure 3(a)). Among them, AC018761.2 (degree = 49), AC090607.4 (degree = 46), and ABALON (degree = 42) were the top 3 DElncRNAs that covered the most DEmRNAs. Then, to investigate the functions of DEmRNAs in shared and osteolysis-specific DElncRNA-DEmRNA coexpression network, DAVID 6.8 was used to perform GO and KEGG enrichment analysis. For DEmRNAs in shared DElncRNA-DEmRNA coexpression network, T cell activation (*p* = 1.53*E* − 02), leukocyte differentiation (*p* = 1.69*E* − 02), leukocyte activation (*p* = 1.77*E* − 02), immune system development (*p* = 2.72*E* − 02), T cell differentiation (*p* = 2.96*E* − 02), and mTOR signaling pathway (*p* = 2.85*E* − 02) were several significantly enriched pathways (Figure 3(b)).

**Figure 4:**
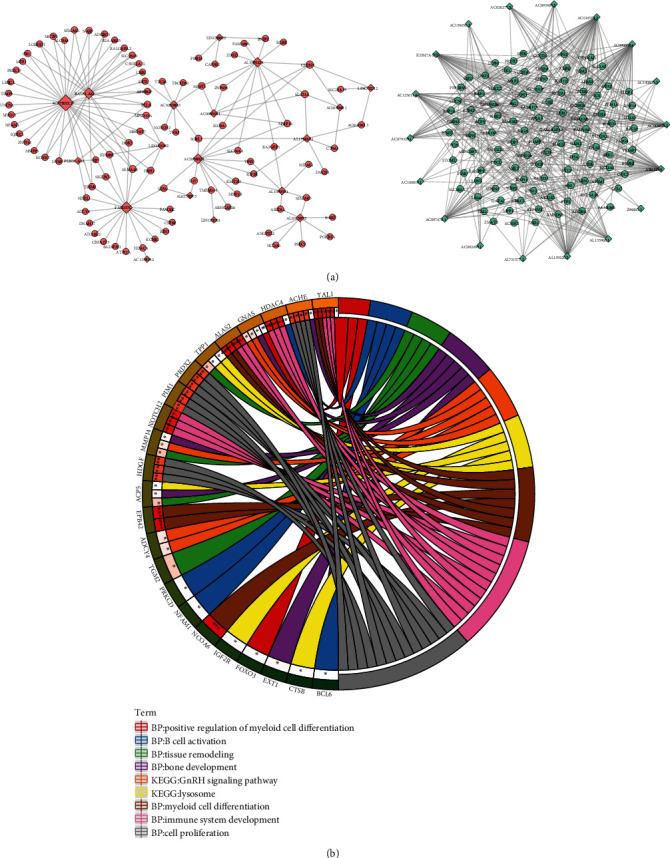
Osteolysis-specific DElncRNA-DEmRNA coexpression network and functional annotation. (a) Osteolysis-specific DElncRNA-DEmRNA coexpression network. The rhombus and ellipses represent DElncRNAs and DEmRNAs, respectively. Red and blue color represent up- and downregulation, respectively. (b) Function enrichment.

A total of 522 osteolysis-specific DElncRNA-DEmRNA coexpression pairs including 36 DElncRNAs and 194 DEmRNAs were obtained (Figure 4(a)). Among them, AC111000.4 (degree = 38), OVCH1-AS1 (degree = 24), and AC016831.6 (degree = 19) were the top 3 DElncRNAs that covered the most DEmRNAs. For DEmRNAs in osteolysis-specific DElncRNA-DEmRNA coexpression network, myeloid cell differentiation (*p* = 4.50*E* − 04), immune system development (*p* = 7.00*E* − 04), cell proliferation (*p* = 2.27*E* − 03), and positive regulation of myeloid cell differentiation (*p* = 4.73*E* − 02) were several significantly enriched pathways (Figure 4(b)).

## 4. Discussion

Osteolytic lesions may develop after THA from a biologic reaction to particulate debris [9]. It is a major complication of THA, causing additional suffering and burden for patients. Aggravating evidence indicates that lncRNAs regulate gene expression via cis- and/or transregulation mechanisms and participate in various biological processes, including chromatin modification, DNA synthesis, cell proliferation, differentiation, and apoptosis [10]. In this present study, we screened out critical genes and lncRNAs correlated with OA and osteolysis by RNA sequencing and bioinformatics analysis.

Due to the close interaction between immune cells and bone cells, immune disorders can lead to abnormal bone metabolism [11]. T lymphocyte differentiation could be made by the “clusters of differentiation” (CD), and the common receptors are CD4 and CD8. CD8, a cell surface glycoprotein, is predominantly expressed on the surface of cytotoxic T killer cells and implicated in the immune system. The CD8 antigen is a specifically recognized antigen displayed by the class major histocompatibility complex I molecules. Long et al. identified a 16-gene biomarker panel to differentiate RA from OA and indicated the lower expression level of CD8A in OA than in RA [12]. Landgraeber et al. reported that the CD4^+^/CD8^+^ ratio was associated with the stage of osteolysis in aseptic loosening [13]. In this study, both CD8A and CD8B were significantly decreased in OA vs. control and osteolysis vs. control. Functional annotation revealed that CD8A and CD8B were enriched in immune-related pathways, such as T cell activation, leukocyte differentiation, leukocyte activation, immune system development, and T cell differentiation. In addition, CD8A was coexpressed with AC111000.4, and CD8B was coexpressed with AC016831.6 in shared the DElncRNA-DEmRNA coexpression network. Taken together, we speculated that AC111000.4 and AC016831.6 may function in the immune process of OA and osteolysis by regulating CD8A and CD8B, respectively.

The FOXO proteins are an evolutionarily conserved family of transcription factors which comprised FOXO1, FOXO3, FOXO4, and FOXO6 in mammals [14]. The four FOXO members share obvious sequence homology and are ubiquitously expressed in various organs, including bone [15]. FOXOs regulate diverse cellular processes, including oxidative stress, metabolism, apoptosis, and inflammation [16]. In addition, FOXOs have been revealed to regulate bone cell functions, osteoblast differentiation, and the maintenance of skeletal homeostasis [17]. Bartell et al. suggested that FOXO3 regulate receptor activator of NF-*κ*B ligand- (RANKL-) induced osteoclast differentiation [18]. Miller et al. reported that FOXO3 play important inhibitory roles in TNF-*α*-mediated osteoclastogenesis and bone resorption [19]. Ambrogini et al. found that overexpression of FOXO3 in osteoblasts may reduce osteoclast numbers [20]. Increased osteoclastogenesis leads to osteolysis. Here, FOXO3 was determined to be a significantly decreased osteolysis-specific DEmRNA and coexpressed with AC090607.4, which indicated the importance of AC090607.4 in osteolysis by targeting FOXO3.

TAL1, also known as SCL, plays a crucial role in hematopoiesis and is causally connected to T cell acute lymphatic leukemia [21, 22]. The fact that embryonic stem cells of Tal1-knockout mice did not develop into osteoclasts *in vitro* supported that Tal1 plays a role in osteoclast differentiation [23]. The maintenance of bone homeostasis mainly depends on the balance between bone-forming osteoblasts and bone-resorbing osteoclasts. The transcription factor TAL1 was of great importance in cell cycle progression and proliferation in differentiating murine bone marrow monocyte precursors [24]. Knockdown of Tal1 in osteoclast progenitors leads to larger osteoclasts, containing more nuclei, and altered expression of a large number genes [25]. Wang et al. found that TAL1 was a differentially expressed transcription factor in osteoporosis [26]. In this study, TAL1 was coexpressed with ABALON in the osteolysis-specific DElncRNA-DEmRNA coexpression network. Therefore, the role of TAL1-BALON in osteolysis should be paid attention.

## 5. Conclusions

In conclusion, we highlighted the roles of four interaction pairs, including two shared lncRNA-mRNA interaction pairs in OA and osteolysis (AC111000.4-CD8A and AC016831.6-CD8B) and two osteolysis-specific interaction pairs (AC090607.4-FOXO3 and TAL1-BALON) in this present work. Taken as a whole, our work made contribution to understanding the pathophysiology of osteolysis. Our study also had a limitation. The sample size for RNA sequencing in this study was small, and further studies with larger sample size are warranted to confirm these results.

## Figures and Tables

**Table 1:** Patient characteristics.

Group	Age (years)	*p* value	Gender	*p* value	BMI (kg/m^2^)	*p* value	Years after THA	Kellgren-Lawrence grade (*n*)	WBC (cells/mL)	*p* value	PLT (cells/mL)	*p* value	ESR (mm/h)	*p* value	CRP (mg/L)	*p* value	Part
Osteolysis	59	0.06	Male	0.64	24.2	0.05	15	—	7.3	0.11	276	0.56	19	0.03	4.2	0.41	Left
	42		Male		25.4		15	—	5.89		210		19		4.2		Right
	70		Female		25.4		17	—	7.42		278		22		9.5		Right
OA	55		Female		30.5		—	3	4.1		225		10		2.1		Right
	40		Female		28.1		—	4	5.77		270		10		2.2		Right
	67		Male		29.8		—	3	5.65		187		10		6.6		Right
Controls	30		Male		26.1		—	—	6.62		233		15		7.7		Left
	29		Male		27.1		—	—	4.45		221		10		2.1		Right
	18		Female		30		—	—	6.85		253		11		1.3		Right

BMI: body mass index; THA: total hip arthroplasty; WBC: white blood cell; PLT: blood platelet; ESR: erythrocyte sedimentation ratio; CRP: C-reactive protein; OA: osteoarthritis.

**Table 2:** Top 10 up- and downregulated DEmRNAs.

Symbol	log_2_FC	*p* value	FDR	Regulation
OA vs. control				
HIST2H4A	2.017903	1.45*E*-12	1.86*E*-08	Up
ZNF778	2.30578	2.34*E*-09	1.50E-05	Up
RAB8A	1.084187	4.61*E*-07	0.001473	Up
MYOM2	3.614517	1.16*E*-05	0.021164	Up
PI3	1.77021	4.16*E*-05	0.05915	Up
VASH1	1.031837	0.000207	0.203576	Up
SNHG28	1.440766	0.000309	0.263506	Up
LILRB3	1.133152	0.000349	0.27883	Up
UPRT	1.107994	0.000428	0.322295	Up
UNC13B	1.407436	0.000467	0.331741	Up
CD8A	-1.49304	2.03*E*-07	0.000865	Down
AL157935.2	-3.11394	2.71*E*-06	0.006946	Down
AL121594.1	-3.12822	4.34*E*-06	0.00926	Down
EPHB4	-2.33741	1.40*E*-05	0.022395	Down
F11R	-1.30037	6.96*E*-05	0.080899	Down
YPEL2	-1.41993	0.000184	0.196205	Down
CHKB-CPT1B	-1.48977	0.00028	0.255821	Down
ZNF80	-1.53824	0.000671	0.393542	Down
EEF1AKMT3	-1.76553	0.000677	0.393542	Down
CD8B	-1.42735	0.000767	0.426828	Down
Osteolysis vs. control				
ZNF778	2.261961	6.77*E*-08	0.000222	Up
LILRB3	1.396428	1.23*E*-06	0.003224	Up
MYOM2	3.476227	3.33*E*-06	0.00575	Up
BCORL1	2.051727	1.40*E*-05	0.018296	Up
RTL5	1.812327	1.85*E*-05	0.022092	Up
FOSL2	1.834524	3.50*E*-05	0.026994	Up
CNTNAP3	2.346929	7.26*E*-05	0.047612	Up
MGAM2	2.714451	9.47*E*-05	0.049807	Up
ERV3-1	1.398861	9.50*E*-05	0.049807	Up
TMEM164	1.378476	0.000107	0.053473	Up
TMEM56-RWDD3	-3.54683	9.81*E*-12	1.29*E*-07	Down
AC013489.1	-4.17415	3.51*E*-06	0.00575	Down
PLVAP	-2.61008	2.39*E*-05	0.026097	Down
PPDPF	-2.88183	2.75*E*-05	0.026397	Down
RPS26	-2.22182	2.82*E*-05	0.026397	Down
HBB	-2.40451	3.29*E*-05	0.026994	Down
RGS6	-2.33305	4.89*E*-05	0.035625	Down
EVPL	-3.08781	5.28*E*-05	0.036452	Down
RIOK3	-2.15799	7.94*E*-05	0.049573	Down
LRRN3	-2.13595	8.98*E*-05	0.049807	Down
Osteolysis vs. OA				
EPHB4	3.187856	9.59*E*-07	0.003135	Up
RASSF5	1.444879	1.47*E*-05	0.023969	Up
FAM118A	1.406201	4.97*E*-05	0.064931	Up
AL354822.1	2.9104	0.000364	0.250326	Up
AL121594.1	2.129941	0.0004	0.259591	Up
ZNF587	1.181929	0.000736	0.320854	Up
SERPING1	1.589099	0.002698	0.608048	Up
LRP1	1.363794	0.004148	0.796847	Up
CASP10	1.098776	0.004527	0.796847	Up
REC8	1.074025	0.004665	0.796847	Up
MTURN	-1.58117	1.09*E*-05	0.021921	Down
HBB	-2.3627	1.17*E*-05	0.021921	Down
CISD2	-1.29668	6.10*E*-05	0.07251	Down
AC093668.3	-2.78122	6.93*E*-05	0.073252	Down
DDA1	-1.3542	8.91*E*-05	0.08323	Down
AC013489.1	-3.94451	0.000133	0.11567	Down
ARL4A	-1.83919	0.00018	0.147286	Down
EPB41	-1.62564	0.000288	0.221659	Down
CR1L	-1.61874	0.000353	0.250326	Down
DLGAP5	-2.34576	0.000426	0.259591	Down

FC: fold change; FDR: false discovery rate; OA: osteoarthritis.

**Table 3:** Top 10 up- and downregulated DElncRNAs.

Symbol	log_2_FC	*p* value	FDR	Regulation
OA vs. control				
NEAT1	5.1159	1.85*E*-18	1.12*E*-14	Up
AL512625.3	2.570376	3.24*E*-05	0.065224	Up
AC016831.6	1.728574	5.90*E*-05	0.088885	Up
AC111000.4	2.064848	0.001215	0.563632	Up
LINC01341	2.02527	0.005032	0.92186	Up
AC018445.3	1.654176	0.010542	0.999047	Up
AC004231.1	1.894872	0.01745	0.999047	Up
AC114752.2	1.692437	0.020267	0.999047	Up
AP003117.2	1.771314	0.023032	0.999047	Up
AC096642.1	1.589585	0.025643	0.999047	Up
AC005726.2	-1.66485	4.64*E*-08	0.00014	Down
PITRM1-AS1	-2.72439	0.000346	0.348099	Down
OVCH1-AS1	-2.18336	0.000714	0.478621	Down
AC005082.1	-2.31905	0.002001	0.670439	Down
AL162457.1	-1.71468	0.002791	0.701277	Down
AC126755.7	-1.50226	0.004787	0.92186	Down
AP006545.1	-1.89154	0.011714	0.999047	Down
AC020659.1	-1.60564	0.013616	0.999047	Down
AC007541.1	-1.69387	0.031639	0.999047	Down
AC068870.2	-1.90393	0.039154	0.999047	Down
Osteolysis vs. control				
FLJ42393	1.62292	7.39*E*-05	0.067195	Up
AC007272.1	2.182036	0.000975	0.279887	Up
AC016831.6	1.690991	0.001091	0.285274	Up
AL359532.1	1.86552	0.001529	0.335756	Up
AL359183.1	1.921891	0.001606	0.340963	Up
AC091185.1	1.772238	0.001807	0.359718	Up
AC018946.1	1.549455	0.002757	0.427746	Up
AC015871.4	1.542564	0.00288	0.427746	Up
AC100788.2	1.613121	0.003089	0.427746	Up
LINC02288	1.673173	0.003444	0.429946	Up
AC123912.4	-3.00991	2.78*E*-10	1.77*E*-06	Down
AC010615.2	-3.72122	1.34*E*-06	0.004253	Down
DDIT4-AS1	-1.59273	1.65*E*-05	0.033738	Down
AC092490.1	-2.67119	2.42*E*-05	0.033738	Down
AC100810.1	-2.2893	5.65*E*-05	0.059947	Down
SLC2A1-AS1	-2.12909	0.000292	0.191562	Down
AC100835.2	-2.25064	0.000369	0.195639	Down
AC097478.1	-2.43982	0.000553	0.242589	Down
LINC00570	-2.3716	0.000559	0.242589	Down
MIR9-3HG	-2.61167	0.000581	0.242589	Down
Osteolysis vs. OA				
AC016737.1	2.007544	0.000286	0.459454	Up
AC124319.2	1.591514	0.003026	0.999986	Up
AL357078.3	1.935801	0.005568	0.999986	Up
AL139020.1	2.119999	0.007539	0.999986	Up
AC004263.1	1.824237	0.015321	0.999986	Up
AC018653.3	1.519922	0.020607	0.999986	Up
AC099521.2	1.851515	0.024567	0.999986	Up
AC110801.1	2.016041	0.0264	0.999986	Up
AL022328.1	1.56358	0.02739	0.999986	Up
AL844908.2	1.529839	0.03055	0.999986	Up
AC123912.4	-3.68915	3.62*E*-08	0.000233	Down
AC010615.2	-3.84396	5.84*E*-06	0.018749	Down
AL356489.2	-3.8055	0.000598	0.654803	Down
AC092821.3	-1.68773	0.001213	0.866219	Down
KRT73-AS1	-1.77092	0.002183	0.999986	Down
FP236383.2	-1.99999	0.002243	0.999986	Down
AL159978.1	-2.32724	0.003423	0.999986	Down
TPM1-AS	-1.77588	0.003634	0.999986	Down
MIR9-3HG	-1.88441	0.004272	0.999986	Down
LINC00570	-1.98324	0.005975	0.999986	Down

FC: fold change; FDR: false discovery rate; OA: osteoarthritis.

## Data Availability

The clinical data used to support the findings of this study are restricted by the Ethics Committee of Shandong Provincial Hospital (No. 2020-123) in order to protect patient privacy. Data are available from Guang Yang (mayayangguang@163.com) for researchers who meet the criteria for access to confidential data.

## Supplementary Materials

Supplementary Materials Figure S1: the volcano plots of DEmRNAs in OA (A), osteolysis (B), OA vs osteolysis (C), DElncRNAs in OA (D), osteolysis (E), and OA vs osteolysis (F). Figure S2: significantly enriched GO terms (A) and KEGG (B) pathways of shared DEmRNAs in OA and osteolysis after THA. Figure S3: significantly enriched GO terms (A) and KEGG (B) pathways of osteolysis-specific DEmRNAs. Figure S4: mTOR signaling pathway and cell cycle were significantly enriched KEGG pathways of shared DEmRNAs (A, B). Hematopoietic cell lineage, cell adhesion molecules (CAMs), porphyrin and chlorophyll metabolism, and systemic lupus erythematosus were significantly enriched KEGG pathways of osteolysis-specific DEmRNAs (C–F).
