# Supplementary material for: Identification of Critical Genes and lncRNAs in Osteolysis after Total Hip Arthroplasty and Osteoarthritis by RNA Sequencing
Source: Biomed Res Int. 2021 Mar 13;2021:6681925. doi: 10.1155/2021/6681925 (PMC7984875; doi:10.1155/2021/6681925)
Supplement: Supplementary Materials — Figure S1: the volcano plots of DEmRNAs in OA (A), osteolysis (B), OA vs osteolysis (C), DElncRNAs in OA (D), osteolysis (E), and OA vs osteolysis (F). Figure S2: significantly enriched GO terms (A) and KEGG (B) pathways of shared DEmRNAs in OA and osteolysis after THA. Figure S3: significantly enriched GO terms (A) and KEGG (B) pathways of osteolysis-specific DEmRNAs. Figure S4: mTOR signaling pathway and cell cycle were significantly enriched KEGG pathways of shared DEmRNAs (A, B). Hematopoietic cell lineage, cell adhesion molecules (CAMs), porphyrin and chlorophyll metabolism, and systemic lupus erythematosus were significantly enriched KEGG pathways of osteolysis-specific DEmRNAs (C–F). [file 6681925.f1.zip › Supplementary Figures (1).docx]

**Supplementary Materials**

**Figure S1** The volcano plots of DEmRNAs in OA (A), osteolysis (B), and OA vs osteolysis (C), and DElncRNAs in OA (D), osteolysis (E), and OA vs osteolysis (F).
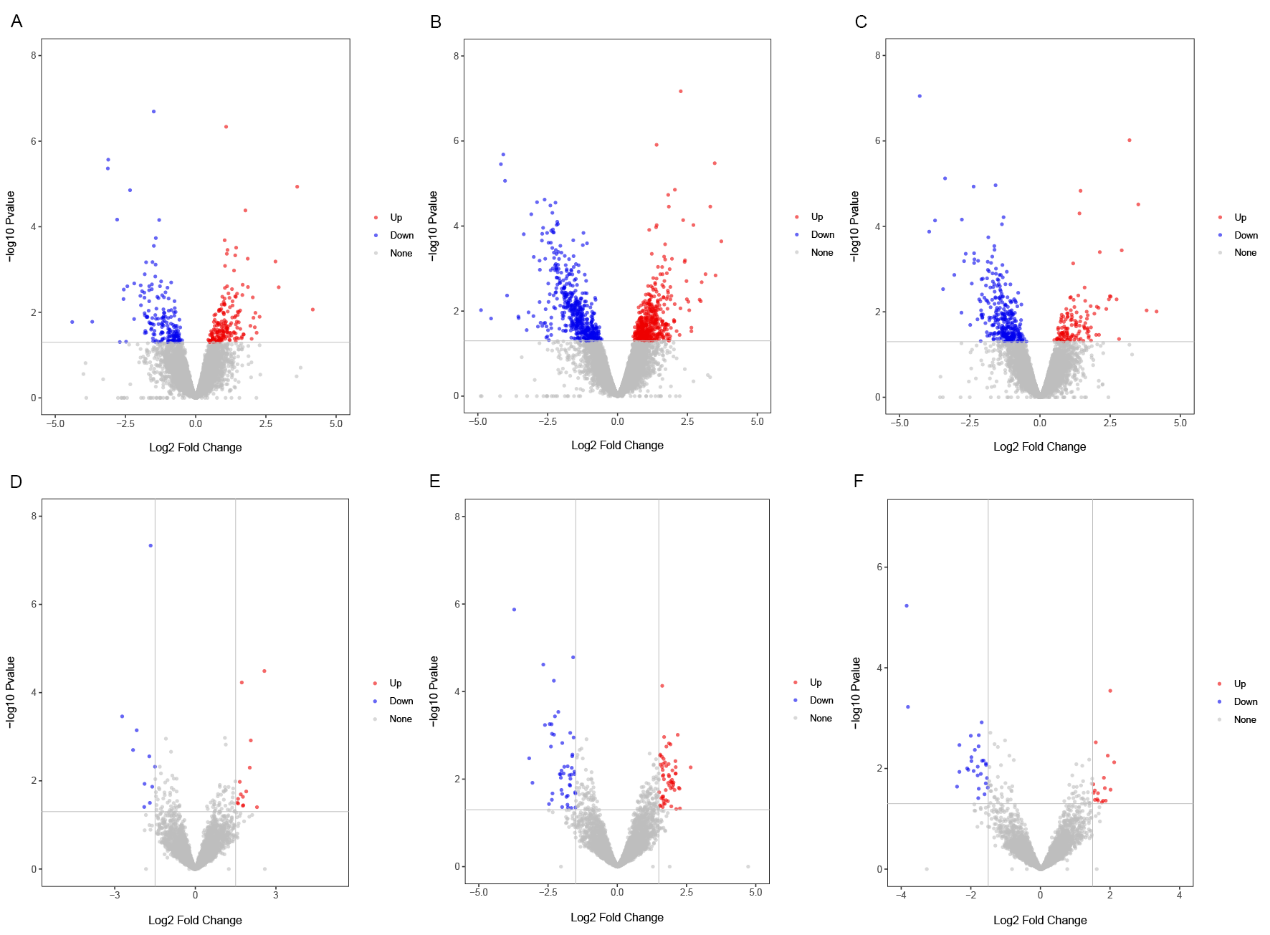


**Figure S2** Significantly enriched GO terms (A) and KEGG (B) pathways of shared DEmRNAs in OA and osteolysis after THA


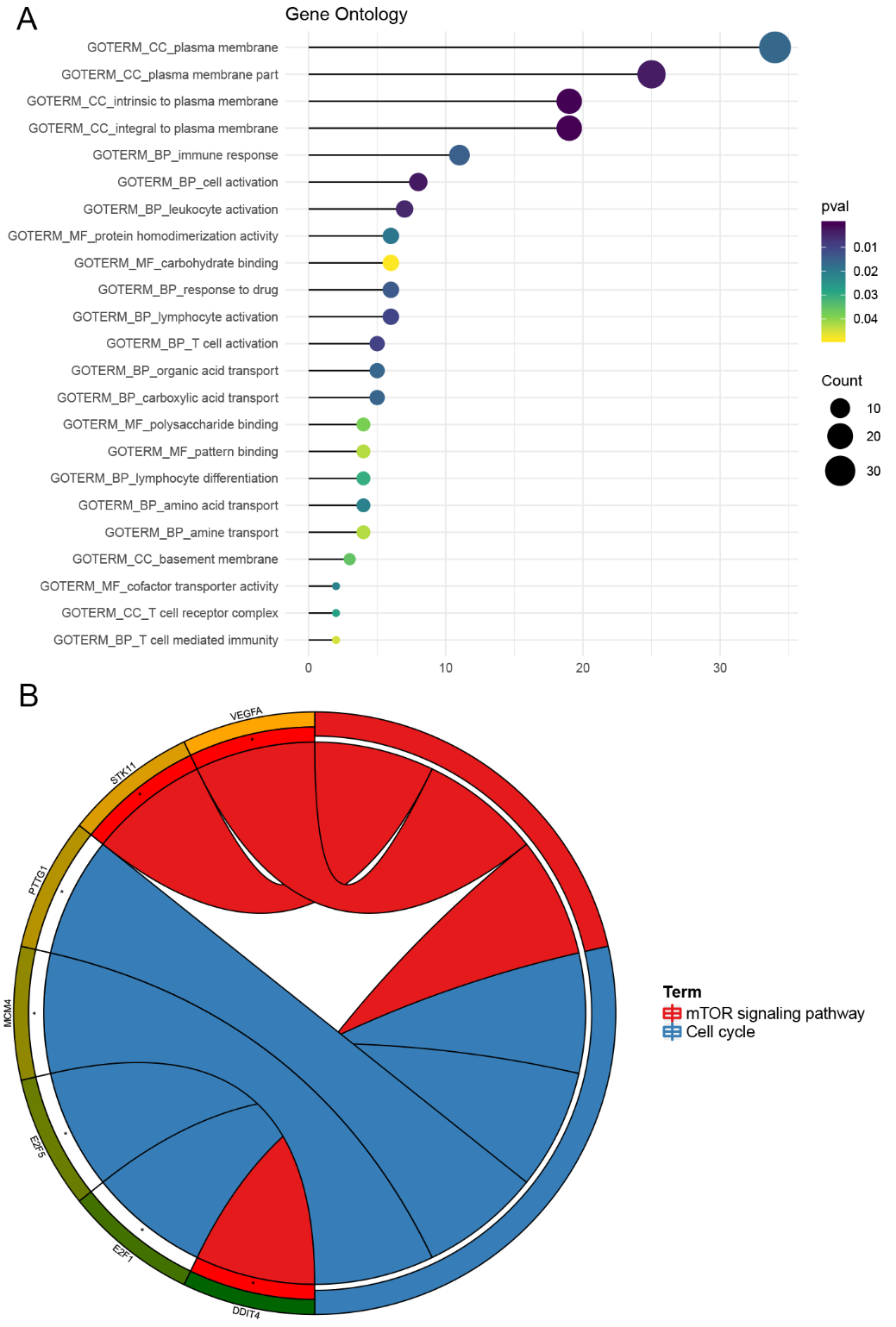


**Figure S3** Significantly enriched GO terms (A) and KEGG (B) pathways of osteolysis-specific DEmRNAs.


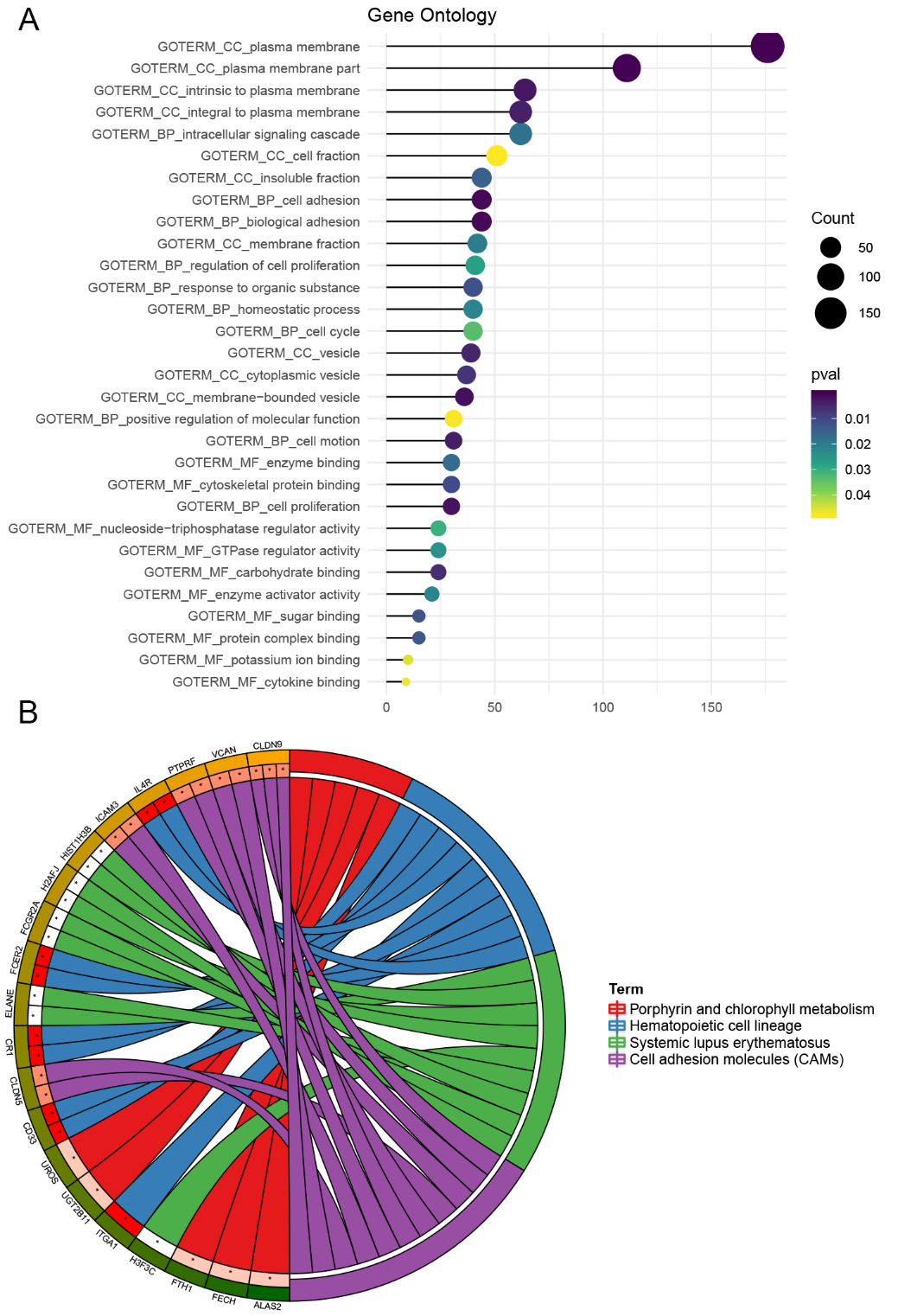


**Figure S4** mTOR signaling pathway and Cell cycle were significantly enriched KEGG pathways of shared DEmRNAs**(**A-B). Hematopoietic cell lineage, Cell adhesion molecules (CAMs), Porphyrin and chlorophyll metabolism and Systemic lupus erythematosus were significantly enriched KEGG pathways of osteolysis-specific DEmRNAs (C-F).


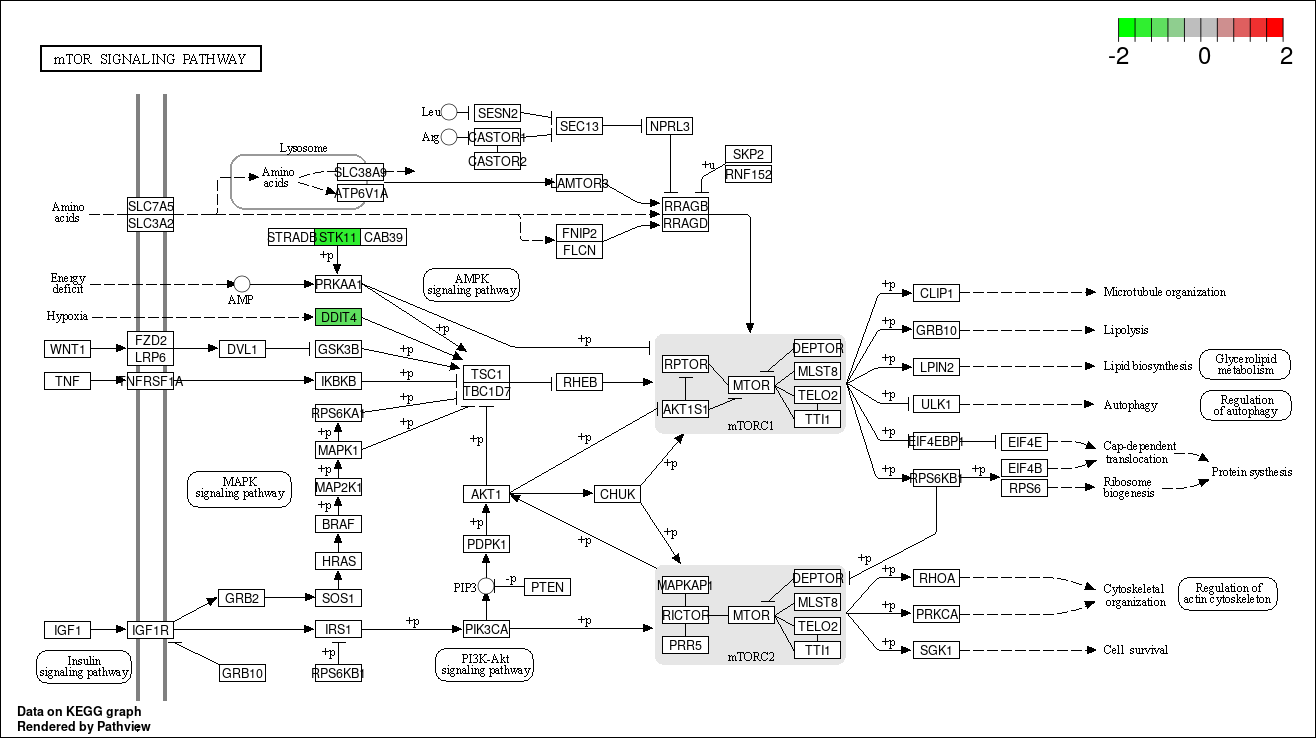


Figure S4 (A)


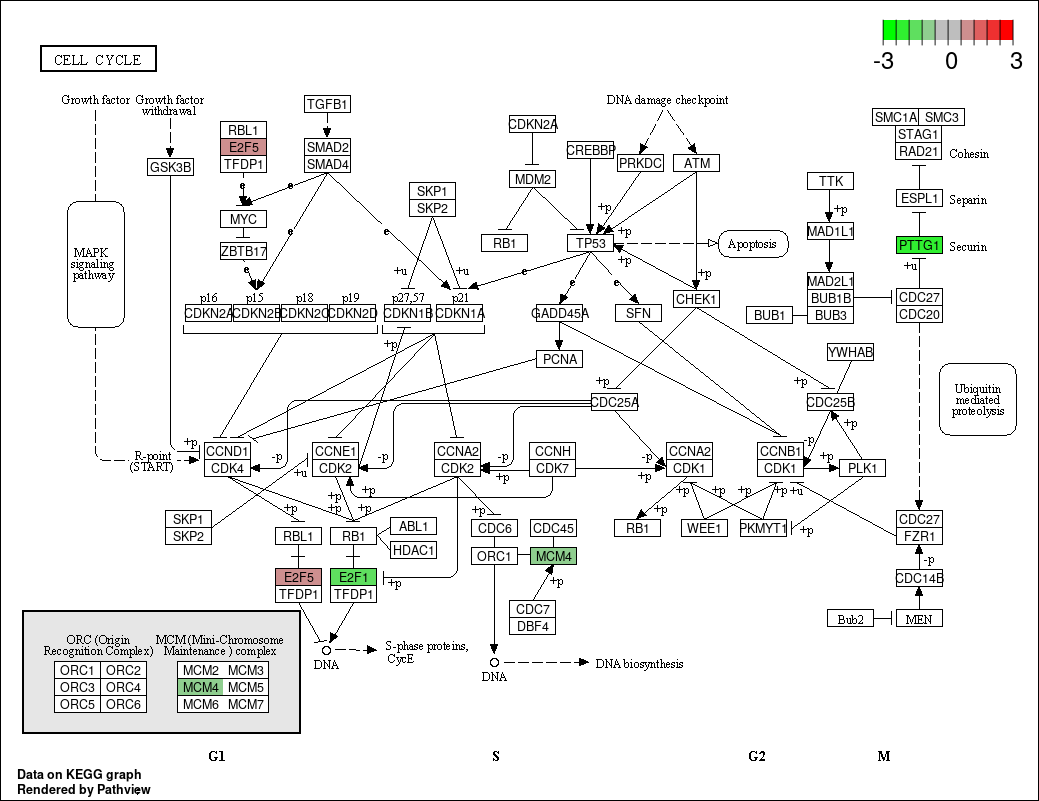


Figure S4 (B)


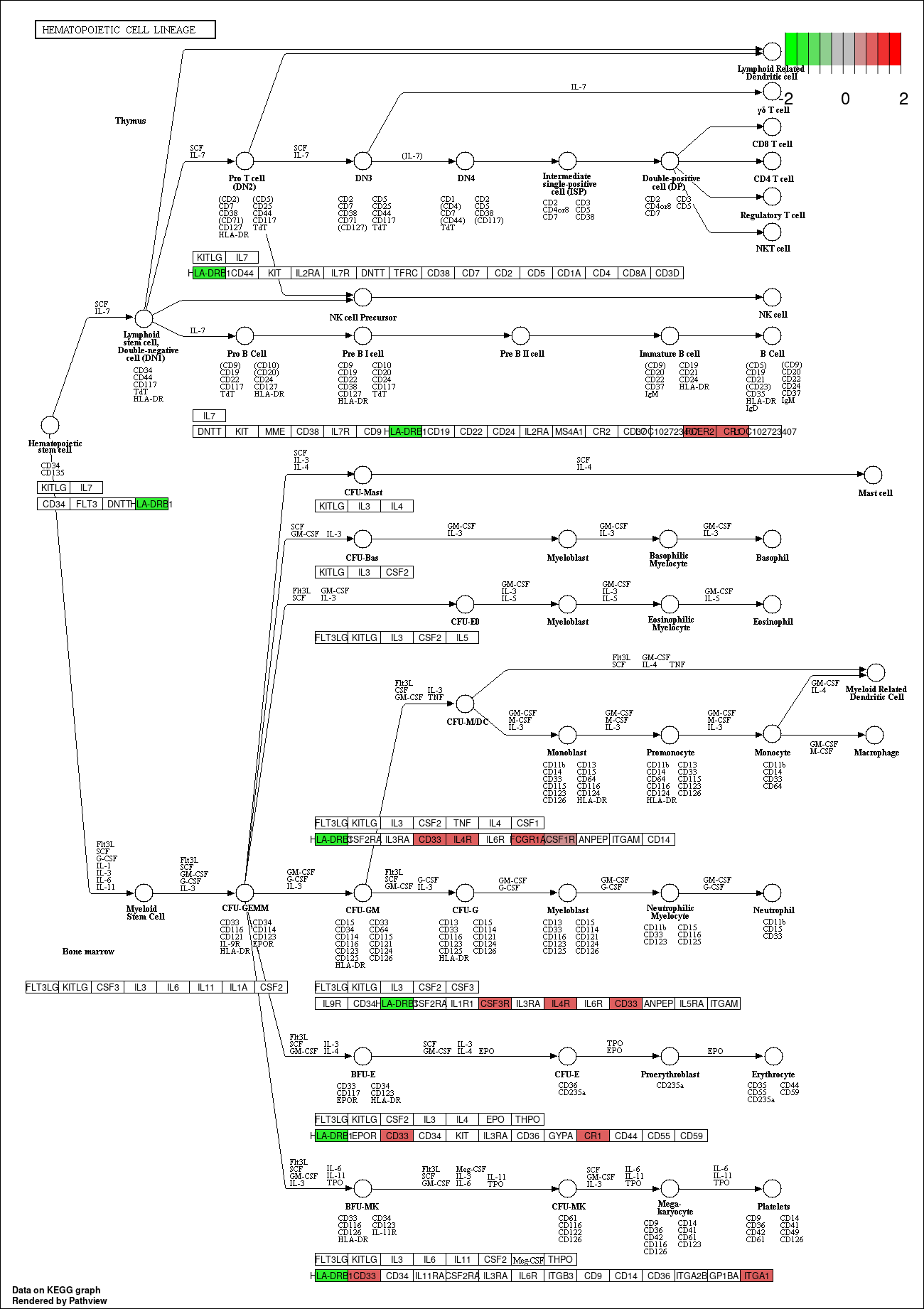


Figure S4 (C)


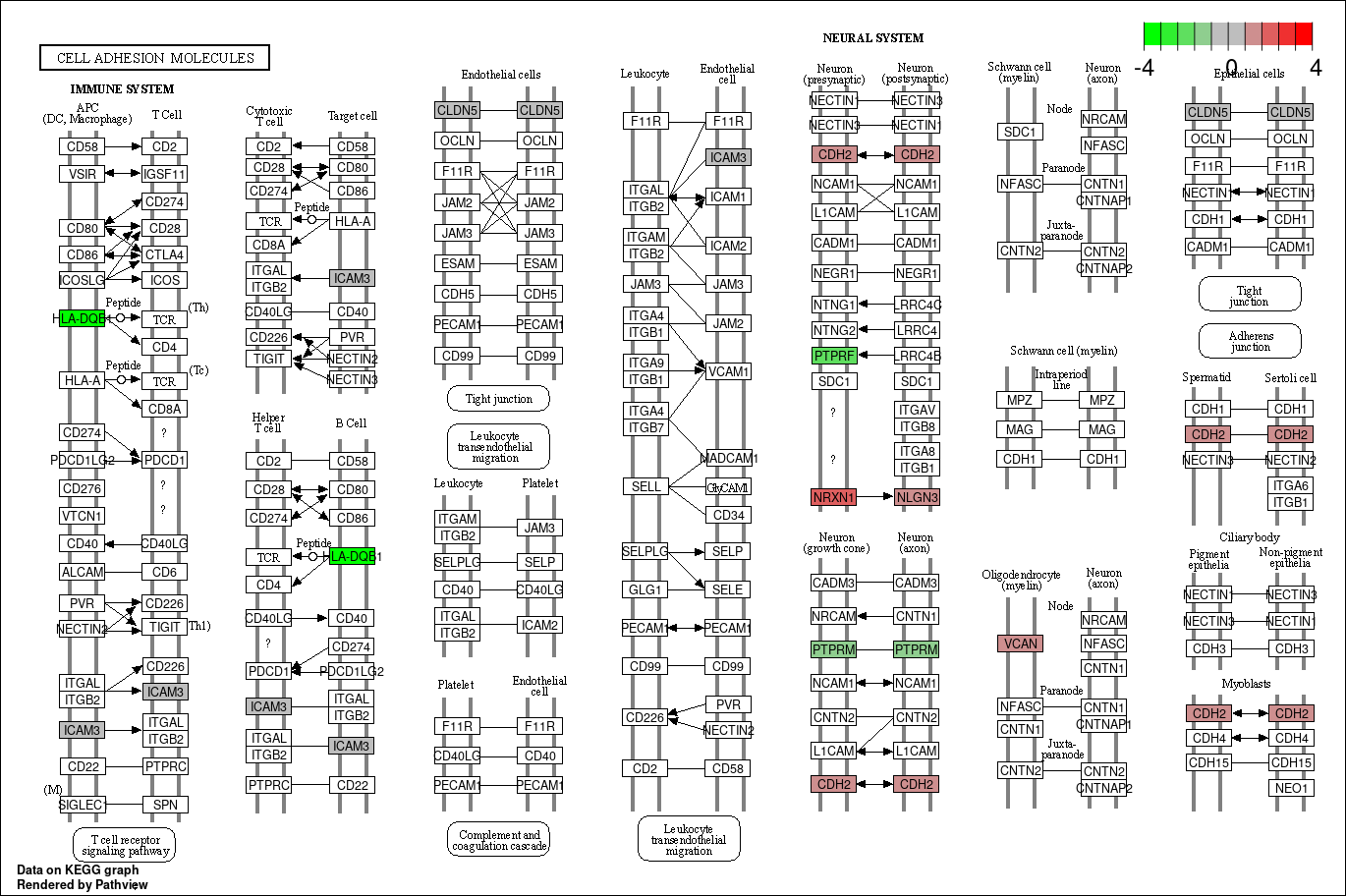


Figure S4 (D)


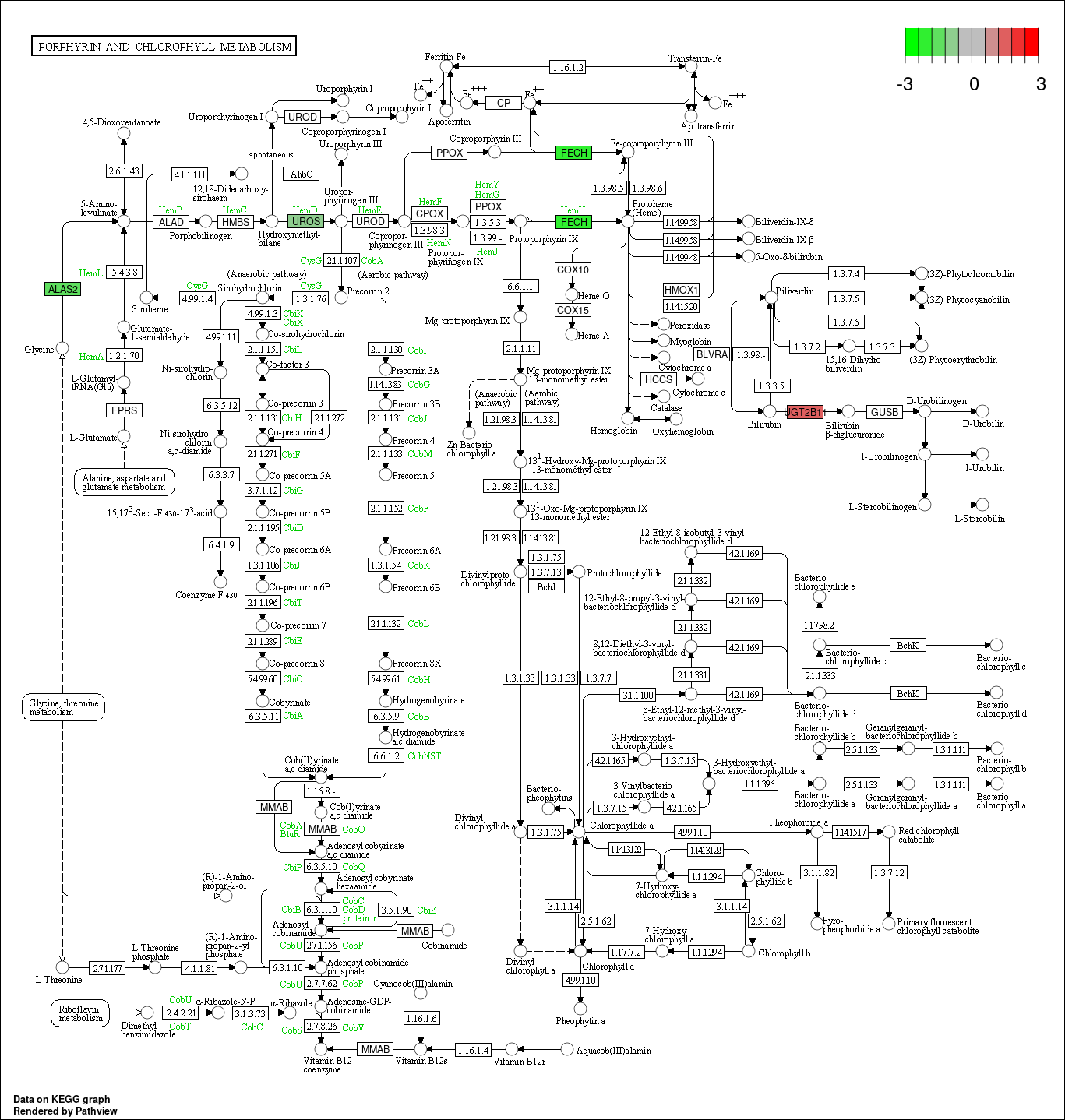


Figure S4 (E)


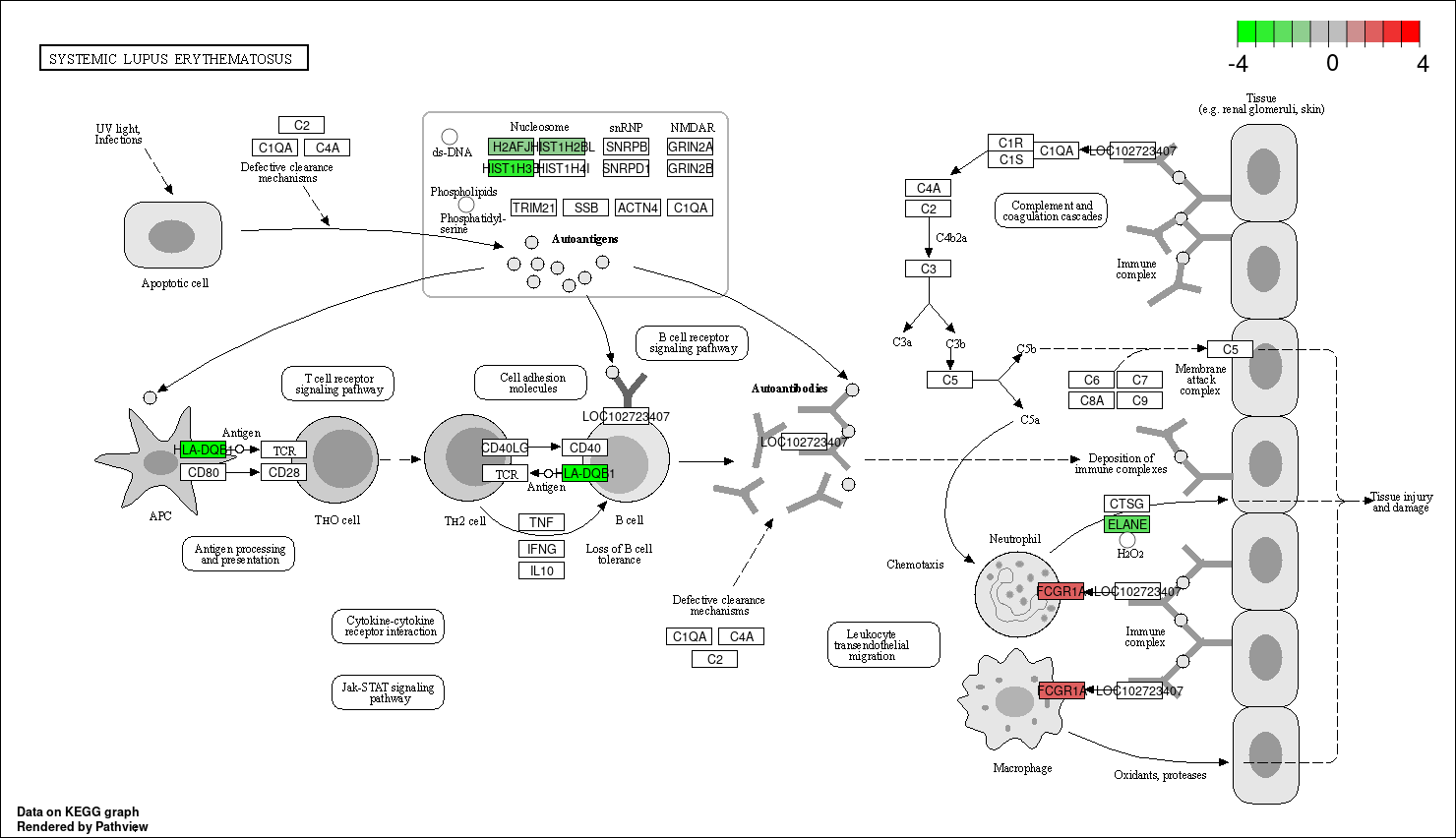


Figure S4 (F)
